# Effects of wild or mutated inoculants on rye silage and its rumen fermentation indices

**DOI:** 10.5713/ajas.19.0308

**Published:** 2019-10-21

**Authors:** Dimas Hand Vidya Paradhipta, Young Ho Joo, Hyuk Jun Lee, Seong Shin Lee, Youn Sig Kwak, Ouk Kyu Han, Dong Hyeon Kim, Sam Churl Kim

**Affiliations:** 1 Division of Applied Life Science (BK21Plus, Institute of Agriculture and Life Science), Gyeongsang National University, Jinju 52828, Korea; 2 Faculty of Animal Science, Universitas Gadjah Mada, Yogyakarta 55281, Indonesia; 3 Department of Crop Science, Korea National College of Agriculture and Fisheries, Jeonju 54874, Korea

**Keywords:** Antifungal, Esterase, Inoculant, Rye, Silage

## Abstract

**Objective:**

This study was conducted to confirm the effects of new inoculants producing-antifungal or esterase substances on rye silage and its rumen fermentation indices by comparing wild with mutated types.

**Methods:**

Rye harvested at dough stage was ensiled into 3 L mini bucket silo (1 kg) for 90 d in triplicate following: distilled water at 20 μL/g (CON); *Lactobacillus brevis* 100D8 (AT) and its inactivation of antifungal genes (AT-m) at 1.2×10^5^ cfu/g, respectively; and *Leuconostoc holzapfelii* 5H4 (FD) and its inactivation of esterase genes (FD-est) at 1.0×10^5^ cfu/g, respectively. After silo opened, silage was sub-sampled for the analysis of ensiling quality and its rumen fermentation indices.

**Results:**

Among the wild type inoculants (CON vs AT vs FD), FD inoculant had higher (p<0.05) *in vitro* digestibilities of dry matter and neutral detergent fiber, the total degradable fraction, and total volatile fatty acid in rumen, while AT inoculant had higher (p<0.05) lactate, acetate, and lactic acid bacteria in silage. Silage pH and the potentially degradable fraction in rumen increased (p<0.05) by inactivation of antifungal activity (AT vs AT-m), but lactate, acetate, and lactic acid bacteria of silage decreased (p<0.05). In silage, acetate increased (p< 0.05) by inactivation of esterase activity (FD vs FD-est) with decreases (p<0.05) of pH, ammonia-N, lactate, and yeast. Moreover, inactivation of esterase activity clearly decreased (p<0.05) *in vitro* digestibilities of dry matter and neutral detergent fiber, the total degradable fraction, and total volatile fatty acid in the rumen.

**Conclusion:**

This study concluded that FD inoculant confirmed esterase activity on rye silage harvested at dough stage, while AT inoculant could not be confirmed with antifungal activity due to the absence of mold in all silages.

## INTRODUCTION

Rye (*Secale cereal L.*) is one of the winter crops used for ruminants to supply the requirement of dietary fiber. Cultivation of rye forage shows a beneficial improvement on soil properties and water quality [1]. Rye has stronger cold tolerance and higher growth rate than other winter forages such as wheat, triticale, and oat [2]. In addition, harvested rye forage at dough stage increases dry matter (DM) yield [3]. However, it could decrease the nutrient digestibility and silage fermentation quality due to increased lignin concentration with reduced water soluble carbohydrate (WSC) content [4].

Application of lactic acid bacteria (LAB) in silage has been widely used to improve the fermentation quality [5] and digestibility of silage [6,7]. Several strains of LAB have abilities of producing antifungal [8,9] and fibrinolytic enzymes [9,10], which could increase the quality of rye silage, especially harvested at the dough stage. An antifungal substance is effective to inhibit the growth of undesirable bacteria [8], whereas fibrinolytic enzymes increase the digestibility of silage by hydrolyzing structural carbohydrate (SC) [11]. In addition, esterase enzymes can break down the lignin linkage in plant cells, which is a problem in late harvested forage [11]. In previous studies, application of antifungal-producing inoculant inhibited mold contamination in silage [9], whereas application of esterase-producing inoculant increased fiber digestibility of silage [10,12].

In our previous study, *Lactobacillus brevis* (*L. brevis*) 100DB and *Leuconostoc holzapfelii* (*Leuc. holzapfelii*) 5H4 were isolated from rye silage and confirmed as antifungal and esterase-producing bacteria, respectively [13,14]. The *L. brevis* 100DB had ability to inhibit mycotoxin-producing fungi such as *Aspergillus*, *Penicillium*, and *Fusarium* species [13], while the *Leuc. holzapfelii* 5H4 was confirmed to produce esterase enzyme by plate assay [14]. Then, mutant types of *L. brevis* 100D8 and *Leuc. holzapfelii* 5H4 were created by inactivated antifungal and esterase excretion genes, respectively.

Therefore, the aim of this study was to confirm the presence of antifungal or esterase activities by both new inoculants on rye silage harvested at dough stage by comparing wild with mutant types, and their effect on rumen fermentation indices.

## MATERIALS AND METHODS

### Inoculant

A complete genome sequence from the wild types of *L. brevis* 100D8 and *Leuc. holzapfelii* 5H4 were determined by PacBio Sequencing. The lanthionine genes of *L. brevis* 100D8 functioned to regulate the production of antifungal, while esterase genes of *Leuc. holzapfelii* 5H4 functioned to regulate the production of esterase enzymes [13,14]. The mutated inoculants were created by knockout of those genes in both LABs using clustered regularly interspaced palindromic repeats (CRISPR) and CRIPR-associated nuclease 9 (Cas9) systems. Each mutant was confirmed by polymerase chain reaction, which confirmed the target gene was disturbed and with reduced the sized amplicons. Mutant type of *L. brevis* 100D8 had no antifungal excretion genes, while mutant type of *Leuc. holzapfelii* had no esterase excretion gene. This procedure to prepare mutated inoculants with its microbial characteristics was presented in our previous study [14].

### Silage production

The rye forage (100 kg) was harvested at the dough stage (27.0% DM) from Gyeongsang National University farm, Jinju, Korea, and then wilted to reach approximately 35.0% DM. After wilting, the rye was chopped into 3 to 5 cm lengths. Prior to ensiling, rye forage was separated into five piles and treated with different inoculants: i) control (CON), applied distilled water at 20 μL/g; ii) wild type of *L. brevis* 100D8 (AT), applied at 1.2×10^5^ colonies forming units (cfu)/g; iii) mutant type of *L. brevis* 100D8 (AT-m), applied at 1.2×10^5^ cfu/g; iv) wild type of *Leuc. holzapfelii* 5H4 (FD), applied at 1.0×10^5^ cfu/g; and v) mutant type of *Leuc. holzapfelii* 5H4 (FD-est), applied at 1.0×10^5^ cfu/g. The doses of inoculant were adjusted based on actual count before ensiling. All inoculants were diluted in sterile pure-ultra distilled water at 20 mL/kg and sprayed onto rye forage. All treatments were packed into 3 L plastic bucket silos (1 kg) and ensiled for 90 d in triplicate at room temperature (20°C). The fresh rye forage (500 g) before ensiling and silage (500 g) after silo opening were sub-sampled for chemical composition and *in vitro* rumen digestibility analyses. Also, silage (20 g) was sub-sampled and blended with 200 mL of sterile ultrapure water for 30 s, and then filtered through two layers of cheesecloth to make silage extraction. The fresh silage extraction was used to analyze pH and microbial counts. After then, silage extraction was stored at −70°C until analyses of ammonia-N, lactate, and volatile fatty acid (VFA).

### Chemical compositions

The sub-sampled forage and silage were dried at 65°C for 48 h and ground to pass 1-mm screen using a cutting mill (Shinmyung Electric Co., Ltd, Gimpo, Korea) for the measurement of chemical compositions and *in vitro* digestibility for 48 h. The DM concentration was determined by drying sample (about 10 g) into the dry oven (OF-22GW, Jeio Tech, Seoul, Korea) at 105°C for 24 h. The crude ash (CA) was determined with a muffle furnace at 550°C for 5 h. The crude protein (CP) and ether extract (EE) were determined by the producers of Kjeldahl (method 984.13; [15]) using N analyzer (B-324, 412, 435, and 719 S Titrino, BUCHI, Flawil, Switzerland) and Soxhlet (method 920.39; [15]), respectively. The neutral detergent fiber (NDF; method 2002.04; [15]) and acid detergent fiber (ADF; method 973.18; [15]) were determined by using Ankom 200 fiber analyzer (Ankom Technology, Macedon, NY, USA). The hemicellulose (HEMI) was determined by calculating the differences between NDF and ADF. The *in vitro* DM digestibility (IVDMD) and *in vitro* NDF digestibility (IVNDFD) were determined after 48 h of incubation by method the Tilley and Terry [16] using an Ankom Daisy (Ankom Technology, USA).

### Fermentation characteristics of silage

Silage pH and ammonia-N were measured using pH meter (SevenEasy, Mettler Toledo, Greifensee, Switzerland) and the colorimetric method described by Chaney and Marbach [17], respectively. The silage extraction was centrifuged at 5,645×*g* for 15 min and collected the supernatant for lactate and VFA analyses. The concentrations of lactate and VFA were determined using HPLC (L-2200, Hitachi, Tokyo, Japan) fitted with a UV detector (L-2400; Hitachi, Japan) and a column (Metacarb 87H; Varian, Palo Alto, CA, USA) according to the method described by Muck and Dickerson [18].

### Microbial counts

Silage extract (first dilution) from 90 d of ensiled silage was continued in several dilutions (10^−5^ to 10^−7^) to determine microbial counts such as LAB, yeast, and mold. The silage extract was plated in triplicate on selective agar medium. The lactobacilli MRS agar media (MRS; Difco, Detroit, MI, USA) was used for LAB count, and potato dextrose agar media (PDA; Difco, USA) for yeast and mold counts. The MRS agar plates were placed in a CO2 incubator (Thermo Scientific, Waltham, MA, USA) at 30°C for 24 h, while PDA plates were incubated at 28°C for 72 h in an aerobic incubator (Johnsam Corp., Boocheon, Korea). Visible colonies were counted from the plates and the number of cfu was expressed per gram of silage.

### *In vitro* rumen incubation

The procedure of animal care was approved by animal ethical committee of Gyeongsang National University, Jinju, Korea. The rumen fluid was collected from two non-pregnant cannulated Hanwoo heifers before morning feeding, their diets consisted of rice straw and commercial concentrate mix at 8:2 ratio plus vitamin-mineral premix. The collected rumen fluid was composited, and then filtered via two layers of cheesecloth. *In vitro* medium was made by mixing rumen fluid with anaerobic culture medium at 1:2 ratio described by Adesogan et al [19]. Dried sample at 0.5 g was put into incubation bottle and 40 mL of *in vitro* medium was added. Then the incubation bottle was gassed with CO_2_ and closed tightly to reach anaerobic condition [19]. Three replications for each treatment were used along with 2 blanks. The bottles were placed into an incubator at 39°C for 0, 3, 6, 12, 24, 48, and 96 h to measure gas pressure. Gas pressure was measured by manometer pressure/vacuum gauge monitor (Fisher Scientific, Traceable, Friendswood, TX, USA) to calculate rumen fermentation kinetics. These kinetics were calculated using nonlinear regression procedure of Statistical Analysis Sofware (SAS) [20] to fit with the model of McDonald [21] following:

$$\text{Y}=\text{A}+\text{B }(1-{\text{e}}^{-\text{c}(\text{t}-\text{L})})\;\text{for t}>\text{L}$$

where A is the immediately degradable fraction; B is the potentially degradable fraction; A+B is total degradable fraction; C is the degradation rate of potentially degradable fraction; L is the lag phase; and t is time of incubation (h).

After incubation, bottles were opened and transferred to 50 mL conical tube to separate remains sample and supernatant (*in vitro* medium) through centrifugation at 2,568×*g* for 15 min (Supra 21k, Hanil Electric Corporation, Seoul, Korea, with rotor A50S-6C No.6). The supernatant was used to analyze ruminal fermentation indices such as pH, ammonia-N, and VFA. The measurement protocols for pH, VFA, and ammonia-N were same as described before.

### Statistical analysis

All collected data were analyzed using one way of analysis of variance through procedure of SAS [20]. The statistical model was *Y_ij_* = *μ*+*T_i_*+*e_ij_*, where *Y_ij_* = response variable, *μ* = overall mean, *T_i_* = the effect of inoculant *i*, *e_ij_* = error term. This statistical model also determined the effect among wild type (CON vs AT vs FD), and the effect among mutant type (AT vs AT-m or FD vs FD-est) to confirm the antifungal or esterase activity in new inoculants. Mean comparison was performed by Tukey’s test. The significant differences were declared at p<0.05.

## RESULTS

### Chemical compositions of rye forage and silage

The concentrations of CP, EE, CA, NDF, ADF, and HEMI of rye forage were 6.48%, 1.49%, 5.13%, 74.4%, 46.2%, and 28.2%, respectively (Table 1). The IVDMD and IVNDFD before ensilage were 50.4% and 38.5%, respectively. After ensiled for 90 d, the inoculant treatments did not affect concentrations of DM, EE, CA, ADF, and HEMI (Table 2). The CP concentration was higher in FD-est silage than CON and AT-m silages (p<0.05; 6.85% vs 6.40% and 6.41%). Silage inoculated with both FD and FD-est produced the lowest NDF concentration, while the highest concentration was with AT-m inoculant (p<0.05; 75.4% and 74.5% vs 77.9%). Silage inoculated with FD produced the higher IVDMD than the other treatments (p<0.05; 45.6% vs 42.2%, 42.4%, 42.5%, and 42.1%). The FD inoculant also produced the highest IVNDFD, while the lowest was by both CON and FD-est inoculants (p<0.05; 35.6% vs 30.6% and 29.6%). Among the wild type inoculants, applied FD inoculant showed higher IVDMD (p = 0.009) and IVNDFD (p = 0.011) than CON and AT inoculants. By inactivation of esterase activity, FD-est inoculant had lower IVDMD (p = 0.005) and IVNDFD (p = 0.008) than FD inoculant. However, AT-m inoculant inactivated antifungal activity had no differences on IVDMD and IVNDFD with AT inoculant.

### Fermentation characteristics of silage

The lowest pH occurred in FD-est inoculant, followed by AT inoculant and AT-m inoculant, while the highest pH was recorded in both CON and FD inoculants (p<0.05; 4.95 vs 5.26 vs 5.53 vs 5.72 and 5.73; Table 3). The concentration of ammonia-N was highest in both CON and AT inoculants, while the lowest was in FD-est inoculant (p<0.05; 0.20% and 0.18% vs 0.10%). The highest lactate concentration was found in AT inoculant, followed by CON, AT-m, and FD inoculants, while the lowest concentration was in FD-est inoculant (p<0.05; 2.08% vs 1.23%, 1.42%, and 1.41% vs 0.73%). The highest acetate concentration was in FD-est inoculant, followed by AT inoculant, while the lowest was in CON (p<0.05; 1.64% vs 0.97% vs 0.61%). The acetate concentration of FD silage was similar to CON and AT-m silages. The concentrations of propionate and butyrate were not detected in the present study. Silage inoculated with FD-est had the lowest lactate to acetate ratio, while the highest ratio was in AT inoculant (p<0.05; 0.44 vs 2.14). Among the wild type inoculants, AT inoculant produced lower pH (p = 0.001), but higher concentrations of lactate (p = 0.004) and acetate (p = 0.023), and lactate to acetate ratio (p = 0.034) than CON and FD inoculant. By inactivation of antifungal activity, AT-m inoculant presented higher pH (p = 0.044), but lower lactate (p = 0.011) and acetate (p = 0.009) concentrations than AT inoculant. While FD-est inoculant inactivated esterase activity had lower pH (p<0.001), concentrations of ammonia (p = 0.001) and lactate (p = 0.017), and lactate to acetate ratio (p = 0.002), but higher acetate concentration (p = 0.004) than FD inoculant.

### Microbial counts of silage

Silage inoculated with AT inoculant produced the highest LAB count compared to all treatments (p<0.05; 7.18 vs 6.40, 6.33, 6.15, and 6.10 log10 cfu/g; Table 4). Yeast count was detected in all treatments, except FD-est inoculant (p<0.05; 6.45, 6.49, 6.56, and 6.20 log10 cfu/g vs not detected). Inactivation of antifungal activity in AT-m inoculant had lower LAB count (p = 0.004) than in AT inoculant, whereas inactivation of esterase activity in FD-est inoculant had lower yeast growth (p<0.001) than in FD inoculant.

### Rumen fermentation kinetics

Degradation of fraction A was not affected by inoculant applications (Table 5). Degradations of fraction B (p<0.05; 8.10 vs 7.00, 7.23, and 7.24 vs 6.01 mL/g) and A+B (p<0.05; 8.32 vs 7.07, 7.34, and 7.39 vs 6.15 mL/g) were highest in FD inoculant, followed by AT, AT-m, and FD-est inoculants, and then in CON. The degradation rate of potentially degradable fraction and lag phase were not affected by selected inoculants. Among the wild type inoculants, silages inoculated with FD produced higher degradations of fraction A (p = 0.012), B (p = 0.008), and A+B (p = 0.007) than CON and AT inoculant. Inactivation of antifungal activity in AT-m inoculant had lower degradation of fraction B (p = 0.037) than AT inoculant, whereas inactivation of esterase activity in FD-est inoculant had lower degradations of fraction B (p = 0.029) and A+B (p = 0.025) than FD inoculant.

### Rumen fermentation indices

Rumen pH and ammonia-N concentration incubated for 96 h were not affected by inoculant applications (Table 6). Total VFA concentration was highest in FD inoculant and the lowest was in CON (p<0.05; 86.8 vs 79.0 m*M*/dL). All silages treated with inoculants presented higher acetate concentration (p<0.05; 65.9%, 66.1%, 66.0%, and 66.6% vs 62.0%) with lower butyrate concentration (p<0.05; 8.91%, 8.69%, 8.95%, and 8.46% vs 10.9%) than CON. The other VFA profiles were not affected by selected inoculants. Among the wild type inoculants, silage inoculated with FD resulted in higher total VFA concentration (p = 0.026) than CON and AT inoculant. Inactivation of antifungal did not affect rumen fermentation indices, while inactivation of esterase activity in FD-est inoculant had lower total VFA concentration (p = 0.005) than FD inoculant.

## DISCUSSION

The chemical composition of rye forage in the present study had higher SC such as NDF, ADF, and HEMI concentrations than rye forage harvested at the heading stage (74.4% vs 60.2%, 46.2% vs 35.9%, and 28.2% vs 24.3%, respectively) [22]. This might be due to the different maturity stage, which was harvested at dough stage in the present study. This previous study also reported that different maturity stage affected the chemical compositions of rye forage [22]. After ensiling, CP concentration was highest in FD-est inoculant due to lower proteolysis activity, which agrees with ammonia-N production in the present study (Tables 2, 3). Kim et al [14] had confirmed that FD and FD-est inoculants produced a combination of cellulase and xylanase enzymes, which could degrade SC during ensiling. This might support that inoculation of FD and FD-est decreased NDF concentration of rye silage in the present study. In agreement, Lynch et al [6] also reported that NDF concentration of alfalfa silage was lower in mixture of cellulase and xylanase treatment than that of control (32.1% vs 33.5%).

Silage inoculated with FD increased both IVDMD and IVNDFD of rye silage in the present study. This was affected by FD inoculant application, which produced the esterase enzyme. In general, esterase enzyme is known to hydrolyze the ester linkage of lignin polymer and increase the accessibility of rumen bacteria and other enzymes to degrade lignin complex [11]. The previous studies also confirmed that application of esterase enzyme or esterase-producing bacteria increased nutrient digestibility in rumen [10,12]. In the present study, no effects of FD-est inoculant on IVDMD and IVNDFD were caused by the inactivation of esterase activity in this inoculant, which decreased degradation of lignin complex in rye silage. Application of AT inoculant in this study confirmed no effects on IVDMD and IVNDFD, which agreed with the results reported by Joo et al [23].

Silage fermentation quality could be affected by the maturity of forage [4]. Especially, the winter forage will be lignified rapidly with reducing WSC content around at dough stage. With that, silage fermentation quality was decreased [5]. In the present study, rye silage harvested at dough stage had higher pH with lower organic acid concentration than rye silage harvested at heading or flowering stages [22]. Among all treatments, the lowest pH in FD-est inoculant was caused by lowest ammonia-N concentration with highest acetate concentration. According to previous studies, it was confirmed that acetate has stronger antifungal activity than lactate [24]. In the present study, higher acetate concentration in FD-est supported the lowest yeast count and ammonia-N concentration in that silage. None of the studies have been conducted with the mutated inoculant on silage. Therefore, it is difficult to explain why FD-est inoculant produced about two times higher acetate than the others. However, some of the other genes in FD inoculant might accelerate the metabolism pathway to produce acetate due to the removal of esterase excretion genes [25]. Among the wild type inoculants, AT as antifungal-producing inoculant had the highest lactate and acetate concentrations, which also promoted the growth of LAB. Inactivation of antifungal activity in AT-m decrease lactate and acetate productions. Antifungal substances can inhibit the growth of yeast and mold during fermentation [8]. However, yeast count was not affected by inactivation of antifungal activity (AT-m vs AT) in the present study. It could be partially supported by higher pH (5.53 vs 5.26) and lower lactate to acetate ratio (1.46% vs 2.14%) between AT-m vs AT, which it did not inhibit the yeast growth effectively [5].

Several studies had confirmed that esterase enzyme improved the degradation of lignin complex and provide more degradable SC [11]. This evidence could indicate that FD inoculant had higher degradation of fraction A, B and A+B compared to CON and all inoculant treatments in the present study. And, it was also supported by an increase of total VFA concentration in the rumen (Table 6) [26]. Inactivation of esterase activity in FD-est inoculant decreased degradable fraction and total VFA in rumen, which also in agreement with decreased IVDMD and IVNDFD in the present study. The silage applied with AT inoculant had highest LAB population in the present study, which the degradable carbohydrates were possibly used more for their growth during ensiling (Table 4). It supported partially that result of AT inoculant had the lowest degradation of fraction A among the wild type inoculants. Inactivation of antifungal activity in AT-m inoculant increased the degradation of fraction B. Several antifungal substances can inhibit the gram positive rumen bacteria [27] and reduce fiber digestion [28], which is in agreement with the present study. Nevertheless, the degradation of fraction B by AT inoculant was still higher than CON.

In general, both wild and mutant type of inoculants showed a higher degradable fraction B or A+B than CON in rumen incubation for 96 h. These results were supported by acetate production in the present study (Table 6), which indicated the high digestibility of SC [29]. Weinberg et al [7] reported that application of LAB with different strains potentially enhanced the rumen digestibility. Contreras-Govea et al [30] also reported that LAB as silage inoculants could alter rumen digestibility, although the improvement on silage quality was limited.

## CONCLUSION

Rye silage inoculated with wild type of *Leuc. holzapfelii* 5H4, an esterase-producing inoculant, had improved nutrient digestibility and rumen fermentation indices of compared to *L. brevis* 100DB. Inactivation of esterase genes in *Leuc. holzapfelii* 5H4 clearly decreased the rumen digestibility and fermentation indices of rye silage. Silage inoculated with wild type of *L. brevis* 100D8 had higher lactate and acetate concentrations than control and *Leuc. holzapfelii* 5H4. Inactivation of antifungal genes in *L. brevis* 100D8 decreased lactate and acetate concentrations of silage but had no effect on yeast count. This study confirmed that wild type of *Leuc. holzapfelii* 5H4 produced esterase activity in rye silage harvested at dough stage, while antifungal activity in *L. brevis* 100DB could not be confirmed due to the absence of mold in all silages.

## Figures and Tables

**Table 1:** Chemical compositions and *in vitro* digestibility of rye forage before ensiled (%, dry matter)

Item	Rye forage
Dry matter	35.0
Crude protein	6.48
Ether extract	1.49
Crude ash	5.13
Neutral detergent fiber	74.4
Acid detergent fiber	46.2
Hemicellulose	28.2
*In vitro* dry matter digestibility1)	50.4
*In vitro* neutral detergent fiber digestibility2)	38.5

1) *In vitro* dry matter digestibility incubated with rumen buffer for 48 h.

2) *In vitro* neutral detergent fiber digestibility incubated with rumen buffer for 48 h.

**Table 2:** Effects of wild and mutated inoculants on chemical compositions and *in vitro* digestibility of rye silage ensiled for 90 d (%, dry matter)

Item	Treatment1)					SEM	p-value		
	
	CON	AT	AT-m	FD	FD-est		CON vs AT vs FD	AT vs AT-m	FD vs FD-est
Dry matter	31.9	32.5	32.2	32.6	32.8	0.919	0.666	0.557	0.871
Crude protein	6.40^b^	6.53^ab^	6.41^b^	6.54^ab^	6.85^a^	0.153	0.602	0.343	0.119
Ether extract	2.88	2.92	2.95	2.95	2.92	0.123	0.859	0.866	0.514
Crude ash	5.92	6.29	5.88	6.31	6.12	0.163	0.018	0.024	0.259
Neutral detergent fiber	76.3^ab^	76.3^ab^	77.9^a^	75.4^b^	74.5^b^	0.691	0.350	0.127	0.079
Acid detergent fiber	47.8	47.9	48.3	47.8	47.0	0.910	0.999	0.612	0.221
Hemicelullose	28.4	28.4	29.6	27.6	27.5	0.767	0.095	0.048	0.935
IVDMD	42.2^b^	42.4^b^	42.5^b^	45.6^a^	42.1^b^	0.750	0.009	0.937	0.005
IVNDFD	30.6^b^	32.4^ab^	32.7^ab^	35.6^a^	29.6^b^	0.962	0.011	0.818	0.008

SEM, standard error of the mean; IVDMD, *in vitro* dry matter digestibility incubated with rumen buffer for 48 h.; IVNDFD, *in vitro* neutral detergent fiber digestibility incubated with rumen buffer for 48 h.

1) CON, without inoculation; AT, wild type of *Lactobacillus brevis* 100D8; AT-m, AT with inactivated antifungal excretion genes; FD, wild type of *Leuconostoc holzapfelii* 5H4; FD-est, FD with inactivated esterase excretion genes.

a,b Means in the same row with different superscripts differ significantly (p<0.05).

**Table 3:** Effects of wild and mutated inoculants on fermentation characteristics of rye silage ensiled for 90 d

Item	Treatment1)					SEM	p-value		
	
	CON	AT	AT-m	FD	FD-est		CON vs AT vs FD	AT vs AT-m	FD vs FD-est
pH	5.72^a^	5.26^c^	5.53^b^	5.73^a^	4.95^d^	0.039	0.001	0.044	<0.001
Ammonia-N (% DM)	0.20^a^	0.18^a^	0.16^ab^	0.17^ab^	0.10^b^	0.029	0.702	0.074	0.001
Lactate (% DM)	1.23^b^	2.08^a^	1.42^b^	1.41^b^	0.73^c^	0.078	0.004	0.011	0.017
Acetate (% DM)	0.61^d^	0.97^b^	0.82^bc^	0.72^cd^	1.64^a^	0.051	0.023	0.009	0.004
Propionate (% DM)	ND	ND	ND	ND	ND	-	-	-	-
Butyrate (% DM)	ND	ND	ND	ND	ND	-	-	-	-
Lactate to acetate	1.97^ab^	2.14^a^	1.46^b^	1.89^ab^	0.44^c^	0.142	0.034	0.085	0.002

SEM, standard error of the mean; DM, dry matter; ND, not detected.

1) CON, without inoculation; AT, wild type of *Lactobacillus brevis* 100D8; AT-m, AT with inactivated antifungal excretion genes; FD, wild type of *Leuconostoc holzapfelii* 5H4; FD-est, FD with inactivated esterase excretion genes.

a–d Means in the same row with different superscripts differ significantly (p<0.05).

**Table 4:** Effects of wild and mutated inoculants on microbial counts of rye silage ensiled for 90 d (log10 cfu/g)

Item	Treatment1)					SEM	p-value		
	
	CON	AT	AT-m	FD	FD-est		CON vs AT vs FD	AT vs AT-m	FD vs FD-est
Lactic acid bacteria	6.40^b^	7.18^a^	6.33^b^	6.15^b^	6.10^b^	0.242	0.004	0.017	0.668
Yeast	6.45	6.49	6.56	6.20	ND	0.188	0.305	0.714	<0.001
Mold	ND	ND	ND	ND	ND	-	-	-	-

SEM, standard error of the mean; ND, not detected.

1) CON, without inoculation; AT, wild type of *Lactobacillus brevis* 100D8; AT-m, AT with inactivated antifungal excretion genes; FD, wild type of *Leuconostoc holzapfelii* 5H4; FD-est, FD with inactivated esterase excretion genes.

a,b Means in the same row with different superscripts differ significantly (p<0.05).

**Table 5:** Effects of rye silages treated with wild and mutated inoculants on rumen fermentation kinetics incubated for 96 h

Item2)	Treatment1)					SEM	p-value		
	
	CON	AT	AT-m	FD	FD-est		CON vs AT vs FD	AT vs AT-m	FD vs FD-est
A (mL/g DM)	0.14	0.07	0.11	0.22	0.15	0.046	0.012	0.386	0.294
B (mL/g DM)	6.01^c^	7.00^b^	7.23^b^	8.10^a^	7.24^b^	0.255	0.008	0.037	0.029
A+B (mL/g DM)	6.15^c^	7.07^b^	7.34^b^	8.32^a^	7.39^b^	0.194	0.007	0.088	0.025
C (%/h)	0.02	0.04	0.03	0.03	0.03	0.007	0.340	0.133	0.374
L (h)	20.3	21.0	21.8	20.5	21.0	0.509	0.497	0.272	0.215

SEM, standard error of the mean; DM, dry matter.

1) CON, without inoculation; AT, wild type of *Lactobacillus brevis* 100D8; AT-m, AT with inactivated antifungal excretion gene; FD, wild type of *Leuconostoc holzapfelii* 5H4; FD-est, FD with inactivated excretion gene of esterase.

2) A, the immediately degradable fraction; B, the potentially degradable fraction; A+B, the total degradable fraction; C, the degradation rate of potentially degradable fraction; L, the lag phase.

a–c Means in the same row with different superscripts differ significantly (p<0.05).

**Table 6:** Effects of rye silages treated with wild and mutated inoculants on rumen fermentation indices incubated for 96 h

Item	Treatment1)					SEM	p-value		
	
	CON	AT	AT-m	FD	FD-est		CON vs AT vs FD	AT vs AT-m	FD vs FD-est
pH	6.39	6.37	6.41	6.41	6.42	0.025	0.313	0.228	0.435
Ammonia-N (mg/dL)	21.7	20.1	20.1	21.0	22.2	1.235	0.189	1.000	0.517
Total VFA (m*M*/dL)	79.0^b^	82.5^ab^	81.4^b^	86.8^a^	81.6^ab^	1.315	0.026	0.638	0.005
Acetate (%)	62.0^b^	65.9^a^	66.1^a^	66.0^a^	66.6^a^	0.510	<0.001	0.308	0.360
Propionate (%)	20.4	20.4	20.7	20.4	20.4	1.154	0.601	0.938	0.389
Iso-butyrate (%)	1.46	1.11	1.06	1.04	1.05	0.227	0.173	0.736	0.173
Butyrate (%)	10.9^a^	8.91^b^	8.69^b^	8.95^b^	8.46^b^	0.218	<0.001	0.647	0.005
Iso-valerate (%)	3.73	2.49	2.31	2.53	2.31	0.561	0.146	0.367	0.325
Valerate (%)	1.51	1.19	1.11	1.08	1.18	0.208	0.148	0.810	0.184
Acetate to propionate	3.04	3.23	3.19	3.23	3.26	0.172	0.187	0.910	0.276

SEM, standard error of the mean; VFA, volatile fatty acid.

1) CON, without inoculation; AT, wild type of *Lactobacillus brevis* 100D8; AT-m, AT with inactivated antifungal excretion gene; FD, wild type of *Leuconostoc holzapfelii* 5H4; FD-est, FD with inactivated excretion gene of esterase.

a,b Means in the same row with different superscripts differ significantly (p<0.05).
